# Sedentary behavior is associated with systemic immune-inflammation index and systemic inflammation response index levels: a cross-sectional analysis of the NHANES 2011–2018

**DOI:** 10.3389/fpubh.2025.1431065

**Published:** 2025-03-19

**Authors:** Xian Wu, Lin Zhong, Yuehong Hu, Lingying Ruan, Kaiyu Zhou, Hanmin Liu, Lina Chen

**Affiliations:** ^1^Key Laboratory of Birth Defects and Related Diseases of Women and Children of MOE, Division of Pediatric Pulmonology and Immunology, NHC Key Laboratory of Chronobiology, West China Second University Hospital, Sichuan University, Chengdu, Sichuan, China; ^2^Key Laboratory of Birth Defects and Related Diseases of Women and Children of MOE, Department of Pediatrics, West China Second University Hospital, Sichuan University, Chengdu, Sichuan, China

**Keywords:** sedentary behavior, systemic immune-inflammation index, systemic inflammation response index, inflammation and immune, NHANES

## Abstract

**Background:**

Sedentary behavior (SB), has been closely linked to numerous detrimental health effects. While the individual and combined impacts of such behaviors on immune-inflammatory responses remain ambiguous, innovative indices like the Systemic Immune-Inflammation Index (SII) and the Systemic Inflammation Response Index (SIRI) are considered as comprehensive tools to assess inflammation. This study endeavors to elucidate the potential correlations between SB, SII, and SIRI, thereby contributing to a deeper understanding of how lifestyle choices influence systemic inflammation profiles.

**Methods:**

This research entailed a retrospective, cross-sectional examination of 39,156 adult participants sourced from 2011 to 2018 of the National Health and Nutrition Examination Survey (NHANES). SASB was used as the independent variable and SII and SIRI as dependent variables. Weighted linear regression was used to assess the correlation between the independent and dependent variables. Smoothed curve fitting and threshold effect analyses were also performed to determine to identify if there was a non-linear relationship between SII and SIRI and SASB. Subgroup analyses were then performed to identify sensitive populations.

**Results:**

A total of 15,789 individuals ≥18 years old were included. Elevated SB levels were correlated with a rise in SII levels in three models (*p* < 0.05). There was a positive correlation of SB and SII (as a continuous variable). At the same, higher SB was associated with increased SIRI level in three models (*p* < 0.05). However, there was a non-linear correlation between SB and SIRI with 485 min (min) being the inflection point.

**Conclusion:**

Among US adults, SII and SIRI exhibited a positive correlation with heightened SB, underscoring the need for more extensive, prospective studies to further elucidate SB’s impact on these inflammation indices.

## Introduction

In recent years, SII and SIRI have received increasing attention from the medical research community as a combination of biomarkers for assessing the systemic inflammation status of individuals (1). SII is an indicator of inflammation by combining the ratio of platelet counts (PLT), neutrophil counts (N), and lymphocyte counts (LYM) in peripheral blood with the formula SII = (PLT × N)/LYM (2, 3), whereas SIRI is based on the N-to-LYM count ratio (NLR) and monocyte (MONO) percentage (4–6), aiming at a more comprehensive assessment of the body’s inflammatory state and immune function balance. These two indices have demonstrated their value in predicting the risk of various chronic diseases (7–9), tumor prognosis (10), and cardiovascular disease progression (11), reflecting not only the local inflammatory response, but also indirectly the systemic inflammatory load and the state of immune function.

Sedentary behavior refers to prolonged periods of time in a sitting or lying position with low energy expenditure while awake, such as prolonged activities like watching television, using a computer, reading, or driving (12). Sedentary activity has become globally prevalent as modern lifestyles have changed, especially in children and adults, where changes in work and learning patterns have led to a significant increase in daily sedentary time. A growing body of research evidence suggests that prolonged sedentary time is closely related to many adverse health outcomes, such as obesity (13), type 2 diabetes (14), cardiovascular disease (15), and certain types of cancer (16). Part of this constellation of health problems may be attributed to the fact that sedentary lifestyles promote the development of a chronic low-grade inflammatory state known as “sedentary disease.”

Given that SII and SIRI serve as valid tools for assessing systemic inflammation, it is particularly important to explore their relationship with SB. Thus, this study planed to explore the relationship between SB and SII and SIRI through cross-sectional analysis of data from NHANES 2011–2018, with the aim of providing new perspectives on understanding the effects of SB on systemic inflammatory status, and providing scientific evidence for developing strategies to reduce sedentary time and improve public health.

## Methods

### Data and sample sources

Information was gathered through the NHANES, an extensive, nationwide, cross-sectional examination aimed at collating data on possible health hazards and nutritional states among non-hospitalized civilians in US. Orchestrated by the National Center for Health Statistics (NCHS), the research employed a sophisticated, multi-phase, categorized, clustered random sampling methodology to ensure a sample accurately mirroring the broader U.S. populace (17). At the same time, the informed consent meticulously secured either from the civilians themselves. Comprehensive details concerning the NHANES study design and its accessible datasets can be found at the official website of NHANES.^1^ Subjects underwent a uniform interview at home followed by a healthcare check at portable assessment facilities for gauging their overall health conditions. Focusing on the link between sedentary habits and levels of SII and SIRI, we examined data spanning seven NHANES waves from 2011 through 2018.

**Figure 1:**
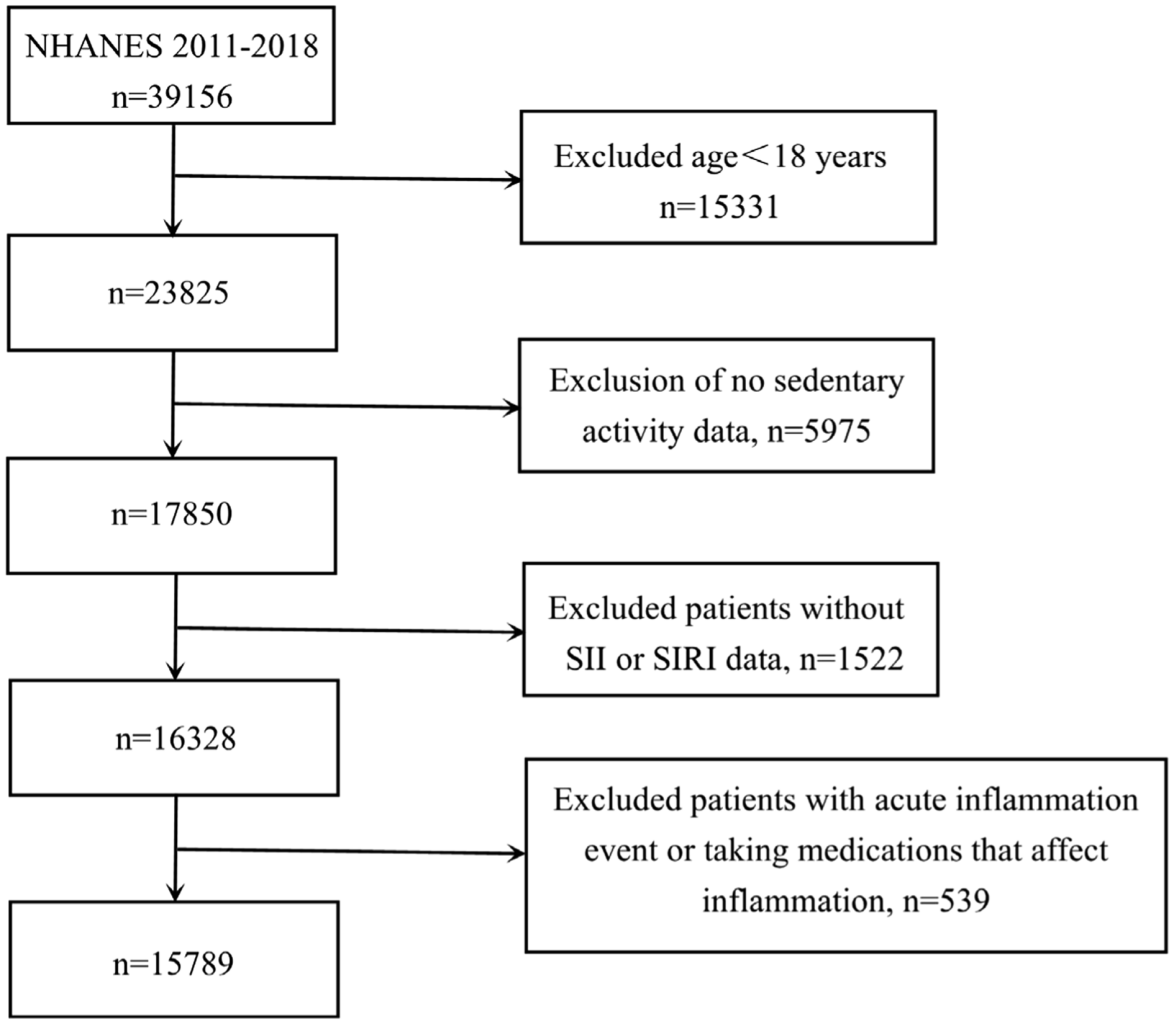
Flowchart of the sample selection from NHANES 2011–2018.

In terms of participant selection, our study excluded individuals who did not meet the following criteria: being 18 years or older, and having complete records on SB, SII, and SIRI measurements, experiencing acute inflammation event and taking medications that affect inflammation. Initially, 39,156 subjects were considered. Following the application of our exclusion parameters: removing those under 18 years old (*n* = 15,331), cases with incomplete data on sedentary habits (*n* = 5,975) or SII and SIRI values (*n* = 1,522), cases with acute inflammation event and taking medications that affect inflammation (*n* = 539) — the final analysis encompassed 15,789 suitable participants (depicted in Figure 1). Based on data available in the NHANES database, acute inflammation was defined as the presence of flu, pneumonia, ear infection during the observation period. Furthermore, medications that affect inflammation include doxycycline, cephalexin, amoxicillin, cefdinir, prednisone, tacrolimus, and other unspecified antibiotics and immunosuppressive agents.

### Definition of SII and SIRI

Hematological assessments, including full blood counts, were conducted using an automated laboratory instrument (Beckman Coulter MAXM). N, LYM, MONO, and PLT were quantified and reported in units of ×10^3^ cells per microliter. Following established methodologies from prior research, SII was derived by multiplying the PLT by the NLR, while SIRI was calculated as the product of the MONO and the same NLR (1, 18). In our analysis, SII and SIRI served as the key exposure variables of interest.

### Assessment of sedentary activity (SB)

In the context of in-home interviews, NHANES participants furnished information on their routine SB via a physical activity questionnaire. Commencing with the 2007–2008 survey wave, NHANES adopted the globally recognized Physical Activity Questionnaire devised by the World Health Organization (WHO) (19), a tool validated across diverse populations with a proven track record for accuracy and consistency (20, 21).

To ascertain the duration of sedentary activities, respondents were queried regarding the typical amount of time they spend seated or reclining on an average day, excluding sleep. This encompassed activities like desk work, commuting by vehicle, reading, television viewing, and computer use. The measure unit used for the sedentary activity is minutes per day (min/day).

### Covariates

In this study, several potential confounding variables, influencing the relationship between SB, SII/SIRI, specifically incorporating demographic factors such as gender, age, race, arm circumference (AC), body mass index (BMI), waist circumference (WC), aspartate transaminase (AST), aspartate aminotransferase (ALT), physical activity, dietary Inflammatory Index (DII), smoking frequency (every day, some days, not at all), stroke (yes/no), diabetes (yes/no), cancer (yes/no), hypertension (yes/no), arthritis (yes/no), high cholesterol (yes/no), asthma (yes/no), and coronary heart disease (CHD) (yes/no).

The Dietary Inflammation Index (DII) is a literature-based tool established by Fan et al. (22) to quantify the inflammatory capacity of diets and has been widely used in the literature to assess the role of diet-induced inflammation in the pathogenesis of a wide range of diseases (e.g., CHD, obesity, diabetes mellitus, and oncologic diseases). Physical activity was assessed through the Physical Activity Questionnaire. This study involved the covariate self-reported PA which was measured by surveys asking participants about the frequency and duration of moderate-intensity and vigorous-intensity physical activity (MVPA) during leisure time, transportation, and occupational activities. Total time spent in MVPA (t-MVPA) of participants per week devoted to moderate- and vigorous-intensity PA was calculated by summing the weekly MVPA and vigorous-intensity (VPA) minutes (23). The “inactive” group was defined as either not performing any MVPA for a week or performing some MVPA for a week but not at the current recommended level (t-MVPA ≥150 min per week). The “active” group was defined as those with MVPA up to the recommended levels.

### Statistical analysis

The continuous data were condensed as mean values alongside standard errors (SE). Differences across tertiles were assessed via weighted ANOVA or Kruskal–Wallis test, with post-hoc corrections. The categorical data were depicted as percentages and weighted chi-square tests were used to assess the differences between categorical ones. Associations between SB and continuous inflammatory markers (SII/SIRI) were analyzed using weighted linear regression, with results expressed as *β* coefficients and 95% confidence intervals.

Any variables were unadjusted in Model 1. Model 2 controlled for three variables including age, gender, and ethnicity. As to Model 3, it further accounted for variables such as high cholesterol, diabetes, asthma, arthritis, CHD, physical activity, DII, angina, stroke, hypertension, cancer, and smoking status. Additionally, subgroups analysis was applied to measure the relationship of SB, SII and SIRI. The stratified factors in subgroups analysis included gender (male/female), age (45<, 45–60, and ≥60 years), BMI (<28 and ≥28 kg/m^2^), AC, WC, hypertension (yes/no), diabetes (yes/no), cancer (yes/no), asthma (yes/no), stroke (yes/no), high cholesterol (yes/no), and CHD (yes/no), enhancing our understanding of these relationships within specific demographic strata. Within our analysis, these segmented factors were considered predefined potential modifiers of effect, with interaction terms incorporated to assess variations in associations across different subgroups. Smooth curve fittings were employed to address the non-linearity. When a non-linear correlation was observed, a two-piecewise linear regression model was used to fit each interval and calculate the threshold effect.

To handle missing data, we imputed median values when the variables were continuous data if the missing proportion was <10%; otherwise, continuous variables were categorized into “unclear groups.” We imputed the most frequent category for categorical variables, based on non-missing observations. All statistical computations were executed utilizing R software (version 3.4.3), complemented by Empower Stats software.

## Results

### Baseline characteristics of participants

The baseline demographic information about minutes of SASB is presented in Table 1. There were totally 15,789 participants enrolled. 46.9% participants (*n* = 7,408) were male and 53.1% participants (*n* = 8,381) were female. SB tertiles 1–3 had SII levels of 524.593 ± 356.596, 555.805 ± 413.183, and 571.243 ± 414.221, and had SIRI levels of 1.278 ± 0.992, 1.437 ± 1.134, and 1.530 ± 1.384. *Post hoc* analysis revealed that Tertile 3 had significantly higher SII than Tertile 1 and Tertile 2 (both *p* < 0.05), and Tertile 3 had significantly higher SIRI than Tertile 1 and Tertile 2 (both *p* < 0.05). Among these SASB tertiles, there were statistical differences in BMI, WBC, N, MONO, ALT, NLR, hemoglobin (HGB), lactate dehydrogenase (LDH), creatinine (Cr), blood urea nitrogen (BUN), cancer, smoke frequency, diabetes, hypertension, stroke, arthritis, high cholesterol, asthma, and CHD (all *p* < 0.05). No significant difference was detected in sedentary time by age and gender. Subjects with longer SB had elevated BMI, AC, WC, Cr, and MONO, and decreased HGB and AST (all *p* < 0.05, Table 1).

**Table 1:** Baseline characteristics of individuals stratified by SB tertiles from NHANES 2011 to 2018.

Minutes of sedentary activity	Tertile 1 (low, *n* = 5,270)	Tertile 2 (middle, *n* = 4,203)	Tertile 3 (high, *n* = 6,316)	*p*-value
Age (years)	47.862 ± 16.946	47.666 ± 18.274	47.427 ± 17.5792	0.437*
BMI (kg/m^2^)	28.066 ± 6.681	28.863 ± 7.466	29.342 ± 8.137	<0.001
AC (cm)	32.182 ± 4.990	32.340 ± 5.290	33.174 ± 5.663	<0.001
WC (cm)	97.834 ± 15.816	98.165 ± 17.230	99.712 ± 17.9387	<0.001
Race				<0.001
Mexican American	970 (18.407%)	420 (10.000%)	524 (8.296%)	
Non-Hispanic White	740 (14.048%)	358(8.506%)	440 (6.967%)	
Non-Hispanic Black	1779 (33.759%)	1914 (45.528%)	3,154 (49.932%)	
Other Race	1781 (33.787%)	1,511 (35.965%)	2,198 (34.805%)	
Gender				0.073
Male	2,443 (46.361%)	1957(46.584%)	3,008 (47.627%)	
Female	2,827 (53.639%)	2,246 (53.416%)	3,308 (52.373%)	
Blood count				
WBC (1,000 cell/uL)	7.291 ± 2.366	7.350 ± 2.542	7.532 ± 2.572	<0.001
LYM (1,000 cell/uL)	2.172 ± 1.211	2.107 ± 1.343	2.121 ± 1.348	0.273
MONO (1,000 cell/uL)	0.562 ± 0.199	0.585 ± 0.217	0.600 ± 0.263	<0.001
N (1,000 cell/uL)	4.302 ± 1.747	4.394 ± 1.787	4.545 ± 1.856	<0.001
HGB (g/dL)	13.767 ± 1.545	13.675 ± 1.550	13.664 ± 1.572	0.119
PLT (1,000 cell/uL)	233.452 ± 64.093	232.721 ± 61.870	232.701 ± 65.116	0.438
SII (1,000 cell/uL)	524.593 ± 356.596	555.805 ± 413.183	571.243 ± 414.221	<0.001
SIRI (1,000 cell/uL)	1.278 ± 0.992	1.437 ± 1.134	1.530 ± 1.384	<0.001
NLR	2.298 ± 1.262	2.308 ± 1.578	2.326 ± 1.753	0.117
ALT (U/L)	25.149 ± 18.607	24.026 ± 16.276	25.272 ± 42.103	0.003
AST (U/L)	25.149 ± 18.607	24.026 ± 16.276	25.272 ± 42.103	<0.001
LDH (IU/L)	131.583 ± 34.548	132.318 ± 28.413	132.222 ± 30.081	0.154
BUN (mmol/L)	5.216 ± 2.320	5.652 ± 2.966	5.718 ± 3.107	<0.001
Cr (mmol/L)	80.590 ± 34.540	89.286 ± 55.935	92.904 ± 73.993	<0.001
Physical activity (n)				0.032
Inactive	2,230 (42.329%)	2089 (49.703%)	3,365 (53.315%)	
Active	3,040 (57.671%)	2,114 (50.297%)	2,951 (46.685%)	
E-DII (n)				0.0024
Anti-inflammatory diet	2,891 (54.862%)	2,159 (51.383%)	3,075 (48.692%)	
Pro-inflammatory diet	2,379 (45.138%)	2044 (48.617%)	3,241 (51.308%)	
Cancer				<0.001
Yes	363 (6.888%)	410 (9.755%)	573 (9.072%)	
No	4,801 (91.101%)	3,626 (86.272%)	5,486 (86.859%)	
Smoke				0.015
Every day	1954 (37.078%)	1,589 (37.806%)	2,234 (35.370%)	
Some days	567 (8.977%)	406 (9.660%)	523 (9.905%)	
Not at all	2,717 (51.556%)	2,206 (52.486%)	3,562 (56.396%)	
Diabetes				<0.001
Yes	635 (12.049%)	506 (12.039%)	869 (13.759%)	
No	4,528 (85.920%)	3,630 (86.367%)	5,261 (83.296%)	
Hypertension				<0.001
Yes	1,694 (32.144%)	1,464 (34.832%)	2,370 (37.524%)	
No	3,593 (68.178%)	2,743 (65.263%)	3,944 (62.244%)	
Stroke				<0.001
Yes	142 (2.694%)	160 (3.664%)	263 (4.164%)	
No	4,966 (94.231%)	3,796 (96.032%)	5,786 (91.609%)	
Arthritis				<0.001
Yes	1,202 (22.808%)	1,062 (25.268%)	1,617 (25.602%)	
No	4,706 (87.0778%)	3,595 (85.534%)	5,267 (83.391%)	
High cholesterol				<0.001
Yes	1,612 (30.588%)	1,395 (33.191%)	2,204 (34.896%)	
No	3,534 (67.059%)	2,685 (63.883%)	3,998 (63.300%)	
Asthma				<0.001
Yes	701 (13.301%)	640 (12.144%)	1,067 (16.894%)	
No	4,566 (86.641%)	3,563 (84.773%)	5,244 (83.027%)	
CHD				<0.001
Yes	136 (2.581%)	160 (3.807%)	297 (4.702%)	
No	4,920 (93.359%)	3,766 (89.602%)	5,679 (89.915%)	

*Comparisons across tertiles were based on weighted ANOVA.

BMI, body mass index; AC, arm circumference; WC, waist circumference; WBC, white blood cell counts; MONO, monocyte counts; N, neutrophil counts; HGB, hemoglobin; PLT, platelet counts; NLR, neutrophil-to-lymphocyte ratio; AST, aspartate transaminase; ALT, aspartate aminotransferase; LDH, lactate dehydrogenase; BUN, blood urea nitrogen; Cr, creatinine; DII, dietary inflammation index; CHD, coronary heart disease.

### The association between SB and increased SII

The findings of the multivariate regression analysis are shown in Table 2. Our findings revealed a positive relationship between elevated SB and heightened SII levels. This link persisted across models: in model 1 (*β* = 9.528; 95% CI: 3.305–15.659; *p* = 0.003), model 2 (*β* = 10.349; 95% CI: 4.341–16.357; *p* = 0.001), and even after full adjustment (*β* = 8.304; 95% CI: 5.382–11.226; *p* = 0.038), signifying that each increment in SB duration corresponded to an average SII increase of 8.304 units (Table 2). The level of SII tended to elevate significantly as the level of SB increased (*p* for trend <0.001).

**Table 2:** Association between minutes sedentary activity, SII and SIRI levels in the participants.

Exposure	Model 1 β (95% CI)	*p*-value	Model 2 β (95% CI)	*p*-value	Model 3 β (95% CI)	*p*-value
SII	9.528 (3.305, 15.659)	0.003	10.349 (4.341, 16.357)	0.001	8.304 (5.382, 11.226)	0.00383
Continuous						
Tertile 1	Reference		Reference		Reference	
Tertile 2	9.478 (−3.522, 22.478)	0.425	11.836 (−1.071 24.743)	0.218	17.031 (9.837, 24.225)	0.003
Tertile 3	19.201(7.172,31.23)	0.002	20.839 (9.240,32.438)	<0.001	19.613 (10.853, 28.373)	0.005
*p* for trend	<0.001		<0.001		<0.001	
SIRI	0.247 (0.134, 0.360)	<0.001	0.239 (0.134, 0.344)	<0.001	0.143 (0.001, 0.285)	0.035
Continuous						
Tertile 1	Reference		Reference		Reference	
Tertile 2	0.071 (0.016, 0.126)	<0.001	0.069 (−0.128, 0.266)	0.732	0.102 (0.009,0.198)	<0.001
Tertile 3	0.078 (0.034,0.122)	<0.001	0.109 (0.075,0.143)	<0.001	0.079 (0.037, 0.121)	0.006
*p* for trend	<0.0001		<0.0001		<0.0001	

95% C1: 95% confidence interval.

Model 1: any variables were unadjusted.

Model 2: three variables including age, gender, and race were adjusted.

Model 3: variables including age, gender, race, BMI, AC,WC, ALT, AST, DII, physical activity, smoke, cancer, hypertension, high cholesterol, diabetes, asthma, arthritis, CHD, angina, and stroke were adjusted.

To validate these findings, SII and SIRI are stratified according to the SB tertiles for sensitivity analysis. Subjects in the highest SB tertile had significantly higher SII than subjects in the lowest SB tertile (*β* = 19.613; 95% CI: 10.853–28.373; *p* = 0.005). Moreover, a marked increase in SII was also observed for the middle SB tertile in comparison to the lowest SB tertile (*β* = 17.031; 95% CI: 9.837–24.225; *p* = 0.003).

**Figure 2:**
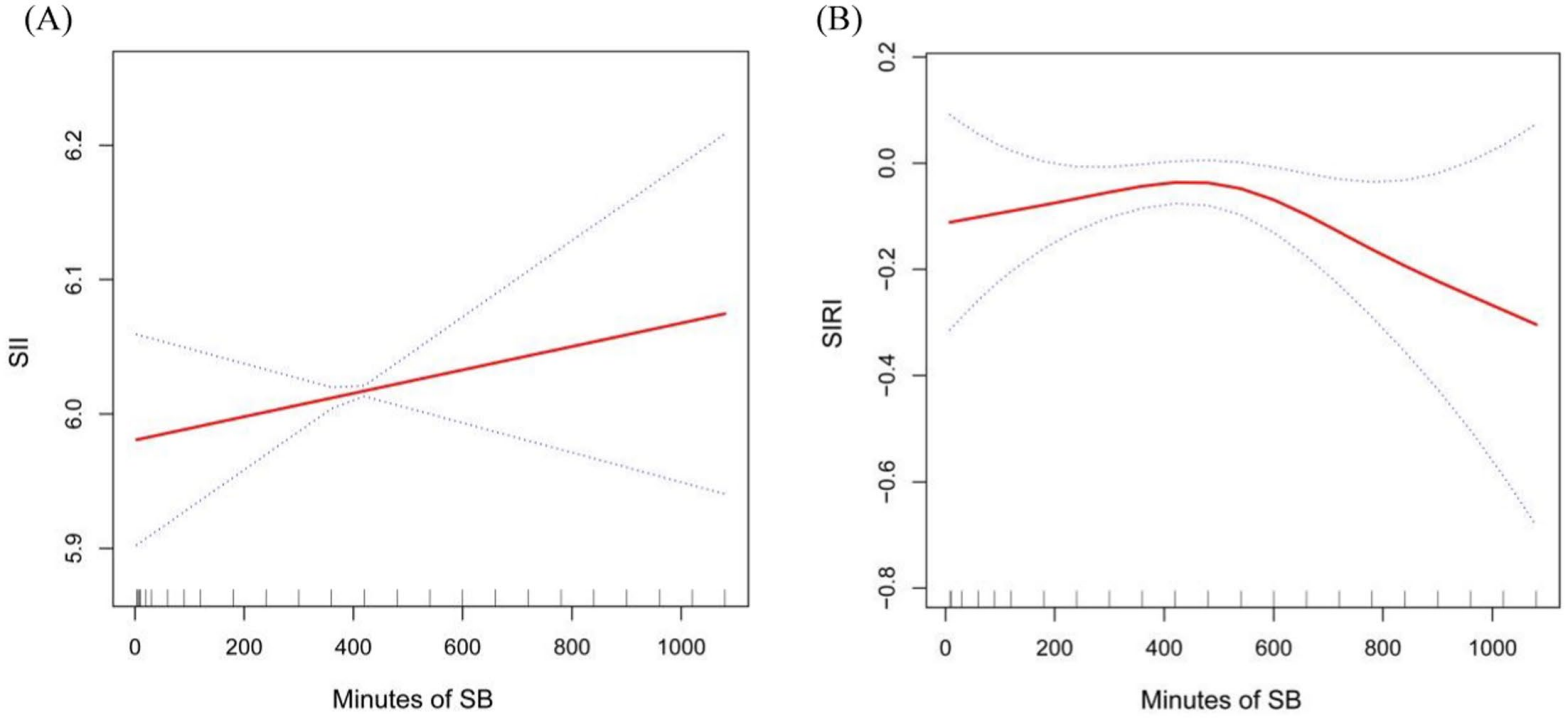
Smooth curve fitting detected the relationship between Sb, SII and SIRI. Panel **(A)** for SII and **(B)** for SIRI.

Graphical representation in Figure 2 further reinforces this relationship, demonstrating a statistically significant positive trend between SB and continuous SII (*p* < 0.05, Figure 2A), highlighting a consistent pattern of SB influencing SII levels.

### The association between SB and increased SIRI

Our findings revealed a connection where higher SB correlated with elevated SIRI levels. This relationship proved significant in model 1 (*β* = 0.247; 95% CI: 0.134–0.360; *p* < 0.001) and persisted with minimal adjustments (*β* = 0.239; 95% CI: 0.134–0.344; *p* < 0.001). Even in model 3 which has comprehensive adjustments, the positive link between SB and SIRI remained, signifying that for every unit increase in SB, the SIRI rises by 0.143 units (*β* = 0.143; 95% CI: 0.001–0.285; *p* = 0.035).

When analyzing tertiles, those in the top SB tertile showed a statistically significant rise in SIRI versus the bottom SB tertile (*β* = 0.079; 95% CI: 0.037–0.121; *p* = 0.006). The middle SB tertile also trended toward higher SIRI levels compared to the lowest SB tertile (*β* = 0.102; 95% CI: 0.009–0.195; *p* < 0.001) (shown in Table 2).

Moreover, we found the potential non-linear relationship between SB and SIRI, adjusting for variables such as sex, age, race, BMI, AC, WC, smoking frequency, history of stroke, cancer, diabetes, CHD, asthma, hypercholesterolemia, hypertension, and arthritis. Our analysis revealed a curved, saturating pattern indicative of non-linearity (*p* for non-linearity = 0.003), as depicted in Figure 2B. Notably, we identified 485 min as the turning point in this relationship. This suggests that, after controlling for various confounding factors, incremental increases in SB up to this point were positively associated with rising SIRI values. Conversely, beyond this inflection point, further increases in SB were inversely related to SIRI, marking a decline in SIRI levels with escalating sedentariness.

### Subgroup analysis

Our subgroup analyses revealed varying associations between SB duration and heightened SII levels (Table 3). Significant links were particularly evident among subgroups categorized by health conditions such as diabetes, asthma, high cholesterol, and arthritis (all *p*-values <0.05). Additionally, notable relationships between SB and SII were observed in subgroups of females, individuals aged over 45 years, those with a BMI exceeding 28 kg/m^2^, higher AC and elevated WC (*p* < 0.05).

**Table 3:** Subgroup analysis of the association between minutes sedentary activity SII and SIRI levels.

Subgroup	*n*	SII		SIRI	
		OR (95%CI)	*p*	OR (95%CI)	*p*
Gender					
Male	7,408	0.0274 (−0.0041, 0.0589)	0.125	0.259 (0.123, 0.393)	<0.001
Female	8,381	0.0680 (0.0303, 0.1057)	<0.001	0.246 (0.117, 0.375)	<0.001
Age (years)					
<45	7,357	0.021 (−0.006, 0.048)	0.160	0.047 (−0.038, 0.132)	0.351
45–60	3,651	0.058 (0.012, 0.104)	0.004	0.232 (0.073, 0.391)	0.002
>60	4,781	0.079 (0.014, 0.144)	0.003	0.611 (0.328, 0.894)	<0.001
BMI (kg/m^2^)					
<28	7,903	0.011 (−0.033, 0.055)	0.557	0.116 (−0.024, 0.256)	0.128
≥28	7,682	0.083 (0.021, 0.145)	<0.001	0.296 (0.180, 0.412)	<0.001
Hypertension					
Yes	5,528	0.056 (0.017, 0.095)	0.004	0.315 (0.103, 0.527)	<0.001
No	10,280	0.029 (0.006, 0.052)	0.029	0.183 (0.074, 0.292)	<0.001
Diabetes					
Yes	2010	0.093 (0.020, 0.166)	0.002	0.432 (0.218, 0.646)	<0.001
No	13,419	0.036 (0.013, 0.059)	0.001	0.174 (0.109, 0.239)	<0.001
Cancer					
Yes	1,346	0.038 (−0.063, 0.139)	0.430	0.581 (0.274, 0.888)	<0.001
No	13,913	0.042 (0.019, 0.065)	<0.001	0.203 (0.157, 0.249)	<0.001
Asthma					
Yes	2,408	0.067 (0.001, 0.133)	0.041	0.332 (0.116, 0.548)	0.004
No	13,373	0.038 (0.014, 0.062)	0.002	0.219 (0.149, 0.289)	<0.001
Stroke					
Yes	565	0.026 (−0.151, 0.203)	0.628	0.172 (−0.402, 0.746)	0.474
No	14,548	0.041 (0.010, 0.072)	0.001	0.227 (0.139, 0.315)	<0.001
High cholesterol					
Yes	5,211	0.048 (0.003, 0.093)	0.034	0.346 (0.117, 0.575)	<0.001
No	10,217	0.034 (0.005, 0.063)	0.027	0.164 (0.063, 0.265)	<0.001
Arthritis					
Yes	3,881	0.061 (0.000, 0.122)	0.031	0.485 (0.254, 0.716)	<0.001
No	13,568	0.040 (0.006, 0.074)	0.006	0.125 (0.033, 0.217)	<0.001
CHD					
Yes	593	0.032 (−0.149, 0.213)	0.655	0.262 (−0.194, 0.718)	0.371
No	14,548	0.043 (0.017, 0.069)	0.001	0.220 (0.134, 0.306)	<0.001
AC (cm)					
Low	4,956	0.035 (−0.008, 0.078)	0.224	0.184 (0.043, 0.325)	0.024
Middle	5,088	0.011 (−0.026, 0.048)	0.482	0.204 (0.072, 0.336)	<0.001
High	5,052	0.056 (0.019, 0.093)	0.002	0.228 (0.116, 0.34)	<0.001
WC (cm)					
Low	4,967	−0.003 (−0.034, 0.040)	0.863	−0.016 (−0.136, 0.104)	0.549
Middle	4,979	0.023 (−0.003, 0.049)	0.270	0.242 (0.147, 0.337)	<0.001
High	4,989	0.052 (0.007, 0.097)	0.021	0.214 (0.130, 0.298)	<0.001

OR, odds ratio.

Regarding the relationship between SB and SIRI, subgroup analyses highlighted significant interactions in groups distinguished by hypertension, diabetes, cancer, asthma, high cholesterol, and arthritis (*p* < 0.05). Furthermore, a significant relationship between SB and SIRI was detected in participants aged over 45 years, BMI exceeding 28 kg/m^2^, and those with a higher WC (*p*-values <0.05), suggesting that these factors might modify the impact of SB on SIRI.

## Discussion

In this cross-sectional study, 15,789 adults were totally included. We observed that subjects with higher SB had higher SII and SIRI levels. Subgroup analyses showed that the significant association between SB duration and elevated SII levels was particularly evident in females, individuals aged over 45 years, those with a BMI exceeding 28 kg/m^2^, higher AC and WC, and subgroups categorized by health conditions such as diabetes, asthma, high cholesterol, and arthritis. Whereas, the significant association between SB and SIRI was more pronounced in groups categorized by BMI exceeding 28 kg/m^2^, hypertension, diabetes, cancer, asthma, high cholesterol, and arthritis. Smoothed curve fitting showed a linear positive correlation between SB and SII, but a non-linear correlation between SB and SIRI, with 485 as the inflection point. In summary, longer sedentary time was associated with higher levels of SII and SIRI, suggesting that SB may be an important environmental factor in promoting a chronic low-grade inflammatory state.

The GPAQ, a global measure of physical activity that has now been validated in adult populations in several countries around the world. The questionnaire is capable of consistently assessing an individual’s level of physical activity (19, 20). Although the GPAQ is a practical tool, it relies on self-report, may have recall bias, and may not be as accurate as direct measures such as accelerometers and pedometers. Yet while direct measurement methods are typically higher in accuracy, there are some practical limitations to their use. Participants need to wear the monitor for long periods of time, which may interfere with their daily activities and lead to poor compliance issues. In addition, researchers need to have expertise and sufficient time to initialize the monitors, perform the study, and process and analyze the data (24). Therefore, in many cases, these direct measures may not be the most feasible option. Therefore, the GPAQ remains valuable as a standardized self-reporting tool in large-scale studies, especially in resource-limited settings.

Although SII and SIRI are newly created indicators of inflammation in recent years and have been used in clinical practice for a short period of time, SII and SIRI have shown excellent predictive ability in several studies, are non-invasive, simple, and low-cost, and have a wide range of clinical applications. N are a traditional indicator of the inflammatory state of the immune system. Circulating MONO can be converted into macrophages to reach solid tissues and participate in immune defense and damage repair processes. In contrast, LYM regulate the immune system by secreting cytokines and cytolytic activity. Platelets play a central role in thrombosis, which is associated with prognosis in cardiovascular disease, among others (25). Therefore, SII, and SIRI may be more credible composite indices of inflammation. Systemic inflammation is responsible for the pathological process of chronic diseases, and SII and SIRI, as a comprehensive inflammatory index, can be a effective and comprehensive marker of the human being’s inflammatory and the immune system state. These two comprehensive inflammatory indices increase the predictive value of various chronic diseases.

Acute inflammation may have a significant effect on systemic inflammatory markers. Therefore, we excluded subjects with acute inflammatory events (e.g., have flu, pneumonia, ear infection) during the study observation period. However, due to the limited nature of the data collected by NHANES but currently available in the NHANES database on acute inflammatory events, such as acute upper respiratory tract infections (except influenza) from other pathogens, acute gastrointestinal infections, acute urinary tract infections, and acute cutaneous infections, the results of the study may have been affected. In addition, drugs that can affect inflammation such as antibiotics, glucocorticoids, immunosuppressants, and other medications may affect SII and SIRI, resulting in false-negative results. Therefore control of medications, especially the confounding factor of medications that affect inflammation, is essential. We searched the NHANES data for information on drug use, and we excluded subjects who used drugs that affect inflammation during the observation period.

After adjusting for confounders, individuals with higher SII or SIRI had an increased likelihood of developing cardiovascular disease, which was associated with higher all-cause mortality (26). Some researchers found SII and SIRI levels were higher in patients with complications of coronary artery disease (CAD) relative to patients without complications, suggesting that SII and SIRI might be used as predictors of CAD complications, and that this indicator was more stable than traditional inflammatory markers such as N-LYM ratio and C-reactive protein (CRP) (27). What’s more, data from another study found that people with elevated SII and SIRI had a greater likelihood of developing stroke, and they similarly observed an increase in all-cause mortality in this condition (28). Our findings that prolonged sedentary activity leads to an increase in SII, SIRI, and thus an increased risk of chronic disease will provide a basis for monitoring SB and subsequently developing countermeasures. It may also provide targets for treatment for those who have to be sedentary due to disability.

The possible mechanisms by which SB correlates with SII or SIRI remain unclear right now. Inflammation is thought to be the “initiator” of chronic diseases, like diabetes, CAD, hypertension, chronic obstructive pulmonary disease (COPD), obesity and hyperlipidemia. High and/or low total white blood cell counts are frequently observed in individuals diagnosed with chronic diseases, suggesting that total white blood cell counts may be associated with metabolic disorders (29). Levels of leukocyte counts appear to be dose-dependent with the amount of exercise and leukocyte counts appear to increase in sedentary adults (30). The mechanisms linking SB to chronic inflammation are complex and may involve multiple physiological pathways. Firstly, prolonged SB leads to decreased lipoprotein lipase (LPL) level in weighing skeletal muscle (legs and core) (31).

Reduction or loss of LPL activity in endothelial cells led to impairment of tissue function in the body, it could reduce the uptake of lipoprotein-derived fatty acids, which can lead to metabolic diseases (e.g., hypercholesterolemia, diabetes, obesity, and most importantly, CHD) (22, 32–34). Secondly, higher SB levels were associated with higher levels of CRP, interleukin 6, tissue plasminogen activator which are the marker of inflammation-induced diseases such as cancer, CAD, COPD, dementia (35). Furthermore, insulin sensitivity decreases within hours of being sedentary, which increases the risk of diabetes with sitting still for a long time (36). Therefore, SB may contribute to the inflammatory response through multiple mechanisms, such as decreasing insulin sensitivity (36), decreasing muscle activity leading to abnormal lipid metabolism (31), and influencing cytokine secretion (35), which in turn affects SII and SIRI levels.

Our study has its own strengths. First, it is the first study to assess the relationship between SB and increases in SII and SIRI. Second, the sample size of our study is relatively large, which increases the credibility of the results. Finally, we adjusted for covariates such as BMI, AC, WC, common biochemical activity indicators, and common chronic diseases, which allowed for more reliable results. However, our study has some drawbacks. First, because of the cross-sectional study design, it was hard for us to determine the causal correlation between SB and SII or SIRI. Second, we concluded that SB was correlated with SII and SIRI, but we were unable to determine whether the length of SB had a predictive value for the inflammatory and immune status of our body. Third, although we adjusted for the many covariates of the appeal, we were unable to completely exclude the influence of other possible covariates on our findings. Furthermore, the GPAQ serves as a standardized self-reporting utility, but it relies on self-reporting, may have recall bias, and may not be as accurate as direct measurement methods (e.g., accelerometers and pedometers). Limited data on acute inflammatory events in the NHANES database may have influenced the study results. Therefore, prospective studies with large sample sizes are still needed to confirm the relationship between SB and SII or SIRI.

## Conclusion

Our findings revealed a positive independent correlation between increased SB and raised SII and SIRI levels, necessitating further validation through extensive, prospective studies.

## Data Availability

The datasets presented in this study can be found in online repositories. The names of the repository/repositories and accession number(s) can be found in the article/supplementary material.

## Footnotes

^1^ https://www.cdc.gov/nchs/nhanes/
